# Development of a multiplex Loop-Mediated Isothermal Amplification (LAMP) for the diagnosis of bacterial periprosthetic joint infection

**DOI:** 10.1371/journal.pone.0302783

**Published:** 2024-05-16

**Authors:** Woong Sik Jang, Seoyeon Park, Ji Hoon Bae, Soo Young Yoon, Chae Seung Lim, Min-Chul Cho

**Affiliations:** 1 Department of Emergency Medicine, Korea University Guro Hospital, Korea University College of Medicine, Seoul, Republic of Korea; 2 Department of Laboratory Medicine, Korea University Guro Hospital, Korea University College of Medicine, Seoul, Republic of Korea; 3 Department of Orthopaedic Surgery, Korea University Guro Hospital, Korea University College of Medicine, Seoul, Republic of Korea; Rutgers Biomedical and Health Sciences, UNITED STATES

## Abstract

**Background:**

Periprosthetic joint infection (PJI) is one of the most serious and debilitating complications that can occur after total joint arthroplasty. Therefore, early diagnosis and appropriate treatment are important for a good prognosis. Recently, molecular diagnostic methods have been widely used to detect the causative microorganisms of PJI sensitively and rapidly. The Multiplex Loop-Mediated Isothermal Amplification (LAMP) method eliminates the complex temperature cycling and delays caused by temperature transitions seen in polymerase chain reaction (PCR) methods, making it faster and easier to perform compared to PCR-based assays. Therefore, this study developed a multiplex LAMP assay for diagnosing bacterial PJI using LAMP technology and evaluated its analytical and clinical performance.

**Methods:**

We developed a multiplex LAMP assay for the detection of five bacteria: *Staphylococcus aureus*, *Staphylococcus epidermidis*, *Streptococcus agalactiae*, *Pseudomonas aeruginosa*, and *Escherichia coli*, frequently observed to be the causative agents of PJI. The method of analytical sensitivity and cross-reactivity were determined by spiking standard strains into the joint synovial fluid. The analytical sensitivity of the multiplex LAMP assay was compared with that of a quantitative real-time PCR (qPCR) assay. Clinical performance was evaluated using 20 joint synovial fluid samples collected from patients suspected of having bacterial PJI.

**Results:**

The analytical sensitivity of the gram-positive bacterial multiplex LAMP assay and qPCR were 10^5^/10^4^ CFU/mL, 10^3^/10^3^ CFU/mL, and 10^5^/10^4^ CFU/mL against *S*. *agalactiae*, *S*. *epidermidis*, and *S*. *aureus*, respectively. For *P*. *aeruginosa* and *E*. *coli*, the analytical sensitivity of the multiplex LAMP and qPCR assays were 10^5^/10^4^ and 10^6^/10^4^ CFU/mL, respectively. The multiplex LAMP assay detects target bacteria without cross-reacting with other bacteria, and exhibited 100% sensitivity and specificity in clinical performance evaluation.

**Conclusions:**

This multiplex LAMP assay can rapidly detect five high-prevalence bacterial species causing bacterial PJI, with excellent sensitivity and specificity, in less than 1 h, and it may be useful for the early diagnosis of PJI.

## Introduction

Periprosthetic joint infection (PJI) is one of the most severe and devastating complications that can occur after total joint arthroplasty (TJA). PJI is associated with high morbidity and is responsible for severe transient or permanent disabilities such as arthrodesis or leg amputation [1, 2]. The incidence of PJI is estimated to be approximately 1% for shoulder and hip TJA and 2% for knee TJA [3]. Recently, as the number of patients receiving TJA has increased due to an aging society, the incidence of PJI has increased [4].

PJI was precisely defined in 2011 by a group of specialists from the Musculoskeletal Infection Society (MSIS). The task group proposed two major and five minor criteria for defining infections following prosthesis implantation [5, 6]. To diagnose PJI, a physical examination must meet one of two major criteria and at least three minor criteria [5]. Bacteria account for 97–99% of the causative agents of PJI. The two most common are gram-positive bacteria, coagulase-negative *Staphylococcus* (CoNS) and *Staphylococcus aureus*. Gram-negative bacteria are less common; however, the two most common are *Escherichia coli* and *Pseudomonas aeruginosa* [7].

PJI is diagnosed based on clinical, serological, and radiographic findings. However, clinical manifestations and serological test results are not always reliable. Preoperative inflammatory markers, such as erythrocyte sedimentation rate (ESR) and C-reactive protein (CRP), can be helpful and are not specific for PJI [8]. Cultures using tissue or synovial fluid are still considered the gold standard for diagnosing PJI. However, this method can be inaccurate because of several factors, such as using an inappropriate medium, short incubation time, loss of microbial load due to storage conditions, or prior antimicrobial therapy [9]. An accurate diagnosis of PJI is essential to avoid unnecessary surgical procedures and inappropriate antibiotic treatments.

Research is being conducted to detect the causative pathogens of PJI using molecular diagnostic tools, such as broad-range 16S rRNA gene polymerase chain reaction (PCR) analysis and next-generation sequencing [1, 10, 11], to overcome the limitations of culture and improve sensitivity. However, these molecular methods have only partially overcome the limitations of culture testing, such as low sensitivity, poor detection of slow-growing bacteria, and long turnaround times. The methods have limitations in routine clinical laboratories, such as the need for expensive equipment, extended reaction times, and well-trained technicians [4].

Loop-mediated isothermal amplification (LAMP) is a well-established isothermal technique used to detect target nucleic acid sequences. The approach is a highly sensitive, low-cost, single-tube method that uses six primers to amplify specific gene regions [12]. Bst DNA polymerase, a strand-displacement DNA polymerase, enables the formation of a loop structure for inner primers, resulting in LAMP’s unique rapid self-priming amplification [13]. The LAMP assay is widely used to detect microbial pathogens [14]. In addition, LAMP has the advantage of multiplexed detection, allowing the simultaneous detection of multiple targets. A diverse range of multiplex Loop-Mediated Isothermal Amplification (LAMP) detection methods has been developed to date, enhancing application and efficiency of this technique [15]. These include the use of fluorescent probes, lateral flow devices, and multi-channel devices for detection. Specifically, the repertoire of multiplex LAMP methods employing probes encompasses methylation-specific LAMP (MS-LAMP) [16], FRET-based assimilating probe-LAMP [17], fluorescence of loop primer upon self-enriching LAMP (FLOS-LAMP) [18], detection of Amplification by Release of Quenching (DARQ) [19], quenching of Unincorporated Amplification Signal Reporters (QUASR) [20], and Molecular Beacon LAMP (MB-LAMP) [21]. Particularly, FRET-based assimilating probe that signal the presence of target DNA by increasing fluorescence upon target amplification, thereby offering precise and sensitive detection in multiplex LAMP assays.

To our knowledge, limited number of studies have developed LAMP based detection for PJI. In this study, we developed a multiplex LAMP assay to detect the five most common bacteria in PJI [4, 22]—*S*. *aureus*, *S*. *epidermidis*, *S*. *agalactiae*, *E*. *coli*, and *P*. *aeruginosa*. We compared our results with quantitative real-time PCR (qPCR) and performed a limit-of-detection analysis to evaluate the analytical performance of the developed multiplex LAMP assay.

## Materials and methods

### American Type Culture Collection (ATCC) standard bacteria strains, clinical samples, and DNA extraction

The analytical and cross-reactivity evaluations of gram-positive and gram-negative bacteria multiplex LAMP assays were performed with standard strains, including *S*. *epidermidis* (ATCC 12228), *S*. *agalactiae* (ATCC 13813), *S*. *aureus* (ATCC 29213), *P*. *aeruginosa* (ATCC 27853), *E*. *coli* (ATCC 25922), *Enterobacter aerogenes* (ATCC 13048), *Enterococcus faecalis* (ATCC 29212), *Streptococcus pneumoniae* (ATCC 49619), and *Klebsiella pneumoniae* (ATCC 13883). Among the ATCC strains stored in our laboratory, we selected strains that were reported to cause PJI with high frequency, including the LAMP target strain, and used them for cross-reactivity studies.

Clinical performance evaluation was conducted using archived joint synovial fluid specimens stored for quality control purposes in the laboratory at Korea University Guro Hospital from October 1^st^ to 15^th^, 2023. All information that could identify the patient, except for the test results, was removed from these archived specimens. Additionally, specimens stored for quality control purposes in the laboratory were used in the study after IRB approval, in accordance with the IRB policy that written consent is waived. This study was approved by the Institutional Review Board of the Korea University Guro Hospital, Seoul, Republic of Korea (IRB No. 2023GR0356). The clinical samples were confirmed to contain bacterial infections by bacterial culture. A total of 20 clinical samples were used in this study, including four *S*. *aureus*, one *S*. *epidermidis*, one *S*. *agalactiae*, and 14 negative samples. All clinical specimens were stored in a -70°C deep freezer and used for LAMP assays without additional culture tests.

For DNA extraction, the AdvanSure™ E3 system (LG Chem, Seoul, Republic of Korea) was used for all samples, following the manufacturer’s manual. Briefly, 200 μL of samples were loaded into the DNA/RNA extraction cartridge, and DNA was eluted in 100 μL of elution buffer. Residual samples and DNA were stored at -70°C.

### LAMP primer design

The LAMP primer sets for *S*. *epidermidis*, *P*. *aeruginosa*, and *E*. *coli* were designed to target conserved regions of *NUT42_08370*, *fecI3* and *malB* gene, respectively. All LAMP primer sets were designed using Primer Explorer software (version 5; Eiken Chemical Co., Tokyo, Japan). We used previously published LAMP primer sets for S. agalactiae and S. aureus [23, 24]. For the multiplex probe design, two types of additional synthetic oligonucleotide sequences were designed and added to the 5′ end of the LB primer of each LAMP primer set. The 5′ end of the multiplex probe was tagged with a fluorescent marker. In a gram-positive bacteria LAMP assay, the 5′ end of *S*. *epidermidis*, *S*. *agalactiae*, and *S*. *aureus* LAMP probes was tagged with FAM, HEX, and CY5. For the gram-negative bacteria LAMP assay, the 5′ end of *P*. *aeruginosa* and *E*. *coli* LAMP probes were tagged with FAM and CY5, respectively. As in previous studies [25, 26], two types of complementary synthetic oligonucleotide sequences tagged with BHQ1 or BHQ2 at the 3′ end, named quencher probes 1 and 2, were used for quenching FAM/HEX and CY5 fluorophore, respectively. All LAMP primers and probes were synthesized by Macrogen, Inc. (Seoul, Korea; 1).

**Table 1:** Gram positive/negative bacteria multiplex LAMP assay primer sets.

Target (gene)		Name	Sequence (5’-3’)	μM	Reference
*S*. *epidermidis* (*NUT42_08370* gene)		SEP_F3	TGGATATGAAGAAAGTGATGC	4	In this study
		SEP_B3	TCTTCAAATAAAGGCATGACG	4	
		SEP_FIP	GCGGAATCATGGTACTGTTACTTTGCTCTCAAATGATTCTTCCCCT	32	
		SEP_BIP	TCCTTTCCAATCAATTGGATCTTGCGTAAATGTGTTTCCCGTTCT	32	
		SEP_LB	/LNA-T//LNA-C//LNA-A//LNA-C/TGAATTTACTCCTGTG	4	
		SEP_LBP	FAM-CGGGCCCGTACAAAGGGAACACCCACACTCCG/LNA-T//LNA-C//LNA-A//LNA-C/TGAATTTACTCCTGTG	6	
*S*. *agalactiae* (*sodA* gene)		SAG_F3	ATATGATGCGCTTGAGCC	4	[24]
		SAG_B3	ACCACCGTTATTGATGACTG	4	
		SAG_FIP	GAGCAGCATTTGCATTAGCAACATATTTTGATGCTGAGACAATGACAC	32	
		SAG_BIP	ACATCCTGAAATTGGAGAAGACTTTTTTCCTGACGAATATCTTCTGGAAT	32	
		SAG_LF	TGCATGGTGCTTATCATGATGT	10	
		SAG_LB	AGGCGCTCTTAGCTGATGT	4	
		SAG_LBP	HEX-CGGGCCCGTACAAAGGGAACACCCACACTCCGAGGCGCTCTTAGCTGATGT	6	
*S*. *aureus* (*nuc* gene)	SAU_F3	GAAGTGGTTCTGAAGATCCAA	4	[23]
	SAU_B3	CCAAGCCTTGACGAACTAA	4	
	SAU_FIP	AGGATGCTTTGTTTCAGGTGTCGATTGATGGTGATACGGTTA	32	
	SAU_BIP	AATATGGTCCTGAAGCAAGTGCGCTAAGCCACGTCCATAT	32	
	SAU_LF	TCTGAATGTCATTGGTTGACCT	10	
	SAU_LB	GAAGTCGAGTTTGACAAAGGTC	4	
	SAU_LBP	Cy5-GTCAGTGCAGGCTCCCGTGTTAGGACGAGGGTAGGGAAGTCGAGTTTGACAAAGGTC	6	
*P*. *aeruginosa* (*fecI3* gene)	PAE_F3	GCTTCCGTGGTTCCGTCTC	4	In this study
	PAE_B3	GGTTGCGGGCGATCTG	4	
	PAE_FIP	TGCCCAGGTGCTTGCGCAGCATGCCTATCAGGCGTTC	32	
	PAE_BIP	GCCGACCTCGCCCAGGATAGCTCGACCGATTGCCG	32	
	PAE_LF	CGCAGCAACTCGCCATG	10	
	PAE_LB	GCCCAGTGGCTGAAATGGC	4	
	PAE_LBP	FAM-CGGGCCCGTACAAAGGGAACACCCACACTCCGGCCCAGTGGCTGAAATGGC	6	
*E*. *coli* (*malB* gene)		ECO-F3	GGCGCGAAAAAAGAAGTGAT	4	In this study
		ECO-B3	AGGTTGGAGAGCGGTTCAT	4	
		ECO-FIP	GGTTTGCGATCCAGCAAATGCGAACCAGCGAGTTAACCAGG	32	
		ECO-BIP	GTGGTCAGCGTCAGCGTGTGCGAGCAAAAATACGCTTGGC	32	
		ECO-LF	CCAGTTGCAGCACTTCCG	10	
		ECO-LB	CGATTGGCCGTACGCTGGTG	4	
		ECO-LBP	Cy5-GTCAGTGCAGGCTCCCGTGTTAGGACGAGGGTAGGCGATTGGCCGTACGCTGGTG	6	
Quencher probe 1			GAGTGTGGGTGTTCCCTTTGTACGGGCCCG-BHQ1	9	
Quencher probe 2			CCTACCCTCGTCCTAACACGGGAGCCTGCACTGAC-BHQ2	9	

LAMP: Loop-mediated isothermal amplification; F3: forward primer; B3: backward primer; FIP: forward inner primer; BIP: backward inner primer; LF: loop forward primer; LB: loop backward primer; LBP: loop backward probe.

### The gram-positive/negative bacteria multiplex LAMP assay

The multiplex LAMP assay for gram-positive and gram-negative bacteria was performed using an ELPIS Biotech LAMP kit (ELPIS Biotech, Daejeon, South Korea). The gram-positive LAMP reaction mixture consisted of 12.5 μL of 2× reaction buffer, 1 μL of *S*. *epidermidis* LAMP primer mix, 1 μL of *S*. *agalactiae* LAMP primer mix, 1 μL of *S*. *aureus* LAMP primer mix, 2 μL of 9 μM quencher probe 1, 1 μL of 9 μM quencher probe 2, 1.5 μL of DNase/RNase-free distilled water, and 5 μL of sample DNA (final reaction volume: 25 μL). The gram-negative LAMP reaction mixture consisted of 12.5 μL of 2× reaction buffer, 1 μL of *P*. *aeruginosa* LAMP primer mix, 1 μL of *E*. *coli* LAMP primer mix, 1 μL of 9 μM quencher probe 1, 1 μL of 9 μM quencher probe 2, 3.5 μL of DNase/RNase-free distilled water, and 5 μL of sample DNA (final reaction volume: 25 μL). Each bacterial LAMP primer mix was composed of 4 μM of two outer primers (F3 and B3), 32 μM of two inner primers (FIP and BIP), 10 μM of forward loop primer (FLP), 4 μM of backward loop primer (BLP), and 6 μM of BLP probe. The LAMP assay was run on a CFX 96 Touch Real-Time PCR Detection System (Bio-Rad Laboratories, Hercules, CA, USA) at 64°C for 30 min.

### Quantitative real-time PCR

The performance of the gram-positive and gram-negative bacterial multiplex LAMP assays for *S*. *aureus*, *S*. *epidermidis*, *S*. *agalactiae*, *P*. *aeruginosa*, and *E*. *coli* strains was compared and evaluated using qPCR. This study used the *S*. *aureus*, *S*. *epidermidis*, *S*. *agalactiae*, *P*. *aeruginosa*, and *E*. *coli* previously reported qPCR primer sets (Table 2) [27–32]. The thermocycling parameters of all qPCR were as follows: initial denaturation at 50°C for 3 min, 45 cycles of denaturation at 95°C for 15 s, annealing and extension with fluorescence detection at 60°C for 30 s.

**Table 2:** Gram-positive/negative bacteria quantitative real-time PCR primer sets.

Target (gene)	Name	Sequence (5’-3’)	μM	Reference
*S*. *epidermidis* (*atlE* gene)	*SEP_atlE*_F	GGAGGAACTAATAATAAGTTAACTG	10	[28]
	*SEP_atlE*_R	GTCATAAACAGTTGTATATAAGCC	10	
	*SEP_atlE*_P	FAM-CTGCTAATCGTGGTGTTGCTCAAATTAAA-BHQ1	10	
*S*. *agalactiae* (*sodA* gene)	SAG_*sip*_F	GTTCCAGCAGCTAAAGAGGAAG	10	[29]
	SAG_*sip*_R	CCGGTGCTACTTTAGCTACTGG	10	
	SAG_*sip*_P	HEX-CACCAGCTTCTGTTGCCGCTGAAACACCAGC-BHQ1	10	
*S*. *aureus* (*nuc* gene)	SAU_*nuc*_F	CACCTGAAACAAAGCATCCTAAA	10	[30]
	SAU_*nuc*_R	CGCTAAGCCACGTCCATATT	10	
	SAU_*nuc*_P	CY5-TGGTCCTGAAGCAAGTGCATTTACGA-BHQ2	10	
*P*. *aeruginosa* (*gyrB* gene)	PAE_*gyrB*_F	GGCGTGGGTGTGGAAGTC	10	[31]
	PAE_*gyrB*_R	TGGTGGCGATCTTGAACTTCTT	10	
	PAE_*gyrB*_P	FAM-TGCAGTGGAACGACA-BHQ1	10	
*E*. *coli* (16s rRNA gene)	ECO_16s_F	CATGCCGCGTGTATGAAGAA	10	[32]
	ECO_16s_R	CGGGTAACGTCAATGAGCAAA	10	
	ECO_16s_P	CY5-TATTAACTTTACTCCCTTCCTCCCCGCTGAA-BHQ2	10	

### Analytical sensitivity tests

For the analytical sensitivity tests of gram-positive and gram-negative bacteria multiplex LAMP assays, all five standard ATCC bacteria (*S*. *aureus*, *S*. *epidermidis*, *S*. *agalactiae*, *P*. *aeruginosa*, and *E*. *coli*) were cultured; a colony count assay confirmed cell concentrations. The five bacteria were spiked into a normal joint fluid (10^7^ CFU/mL) and serially diluted 10-fold with normal joint fluid from 10^7^ to 10^0^ CFU/mL. DNA was extracted from the samples using an AdvanSure E3 system (LG Chem, Seoul, Republic of Korea). The analytical sensitivity of the gram-positive and gram-negative bacterial multiplex LAMP assays were compared to the qPCR assay. All tests were repeated three times and the minimum concentration was determined as the concentration at which all three tests were positive.

## Results

### Temperature optimization of the gram-positive/negative bacteria multiplex LAMP assay

**Fig 1:**
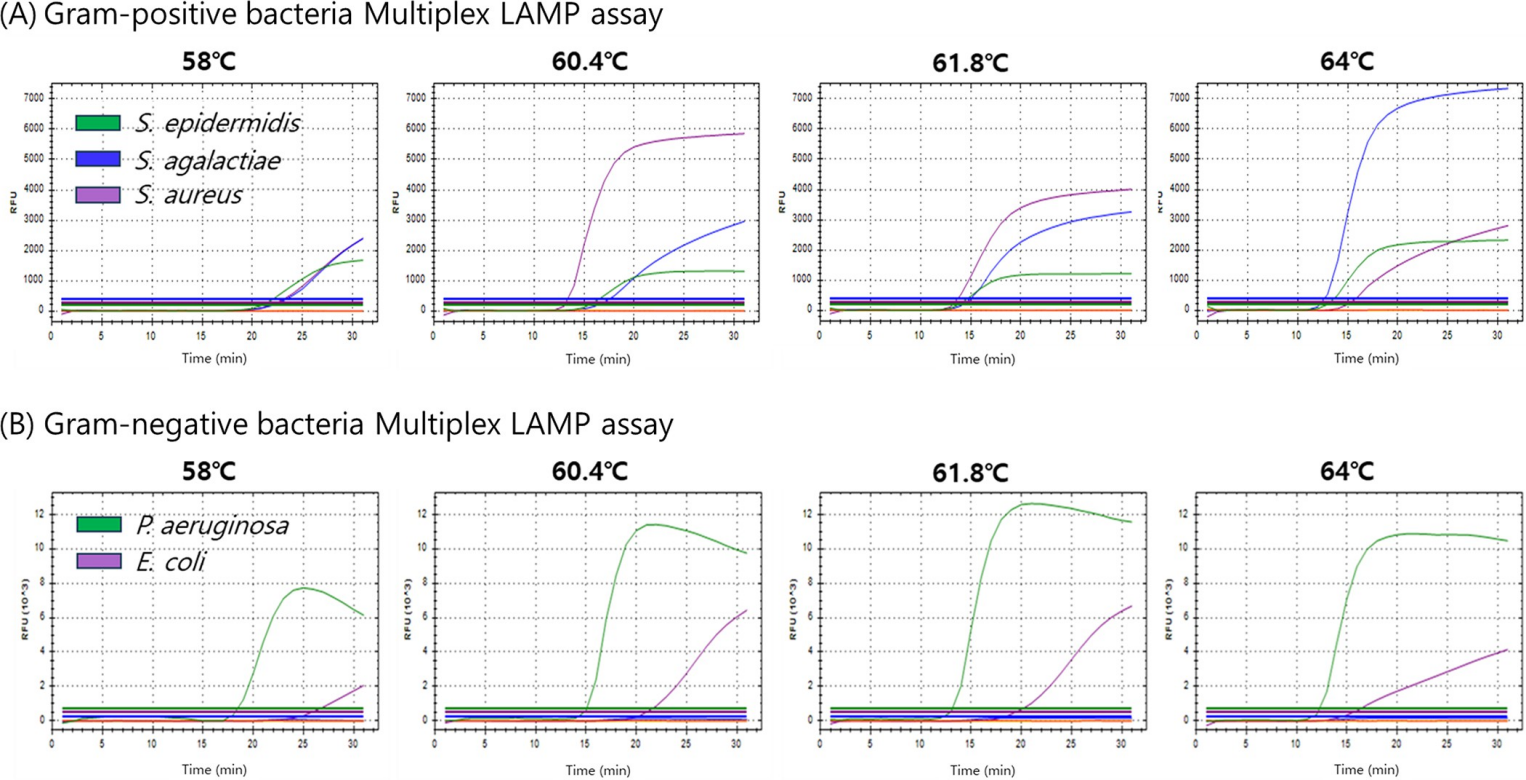
Temperature optimization for the gram-positive/negative bacteria multiplex LAMP primer set. A temperature gradient from 58 to 64°C was performed. (A) The Multiplex LAMP assay for gram-positive bacteria was designed to detect three bacterial species, namely, *S*. *epidermidis* (FAM), *S*. *agalactiae* (HEX), and *S*. *aureus* (CY5). In parallel, (B) the Multiplex LAMP assay for gram-negative bacteria was designed to target two bacterial species, *P*. *aeruginosa* (FAM) and *E*. *coli* (CY5).

Temperature gradient tests (58–64°C) were conducted to determine the optimal temperature for the gram-positive and gram-negative bacteria in multiplex LAMP assays. The gram-positive bacteria multiplex LAMP assay was tested with DNA samples extracted from joint fluid samples spiked with ATCC *S*. *agalactiae*, *S*. *epidermidis*, and *S*. *aureus* (at a concentration of 10^7^ CFU/mL in a 1:1:1 ratio). In contrast, the gram-negative bacteria multiplex LAMP assay was tested with DNA samples extracted from joint fluid samples spiked with ATCC *P*. *aeruginosa* and *E*. *coli* (at a concentration of 10^7^ CFU/mL in a 1:1 ratio) (Table 3; Fig 1). Among the four temperatures assessed (64°C, 61.8°C, 60.4°C, and 58.0°C), both the gram-positive and gram-negative multiplex LAMP assays exhibited the lowest Tt values across all fluorescence channels at 64°C, except for the *S*. *aureus* signal, which was delayed slightly. However, the Tt values for signals from other bacterial strains were considerably delayed at other temperatures. Therefore, based on the overall results, the optimal temperature for conducting multiplex LAMP analysis of gram-positive and gram-negative bacteria was 64°C.

**Table 3:** Temperature optimization for gram-positive/negative bacteria multiplex LAMP assay.

Temp (°C)	Gram-positive bacteria multiplex LAMP assay (Tt values)						Gram-negative bacteria multiplex LAMP assay (Tt values)			
	*S*. *epidermidis*		*S*. *agalactiae*		*S*. *aureus*		*P*. *aeruginosa*		*E*. *coli*	
	Tt	RFU	Tt	RFU	Tt	RFU	Tt	RFU	Tt	RFU
64.0°C	13.1	2307	12.7	7296	15.4	2622	12.2	10662	15.6	3744
61.8°C	13.9	1209	15.0	3171	13.5	3955	13.6	11815	19.4	5935
60.4°C	15.58	1312	17.5	2724	13.2	5819	15.1	10197	21.0	5490
58.0°C	21.1	1576	23.4	1910	22.5	1938	18.5	6830	25.8	1432

LAMP: Loop-mediated isothermal amplification, RFU: relative fluorescence unit, Tt: threshold time.

### Analytical sensitivity of the gram-positive/negative bacteria multiplex LAMP assay

Using the analytical sensitivity test for five strains: *S*. *epidermidis*, *S*. *agalactiae*, *S*. *aureus*, *P*. *aeruginosa*, and *E*. *coli*, the analytical performance of the multiplex LAMP assay for gram-positive and gram-negative bacteria was evaluated and compared with that of the qPCR assay (Table 4). Each DNA sample extracted from the joint fluid spiked with ATCC bacteria, diluted 10-fold in seven levels, was used for analytical sensitivity testing of the gram-positive and gram-negative bacteria multiplex LAMP and qPCR assays. The analytical sensitivity of the gram-positive bacterial multiplex LAMP assay and qPCR were 10^5^/10^4^ CFU/mL, 10^3^/10^3^ CFU/mL, and 10^5^/10^4^ CFU/mL against *S*. *agalactiae*, *S*. *epidermidis*, and *S*. *aureus*, respectively. For *P*. *aeruginosa* and *E*. *coli*, analytical sensitivities of the multiplex LAMP assay and qPCR were 10^5^/10^4^ CFU/mL and 10^6^/10^4^ CFU/mL, respectively. In all test samples, except for *S*. *epidermidis*, the gram-positive and gram-negative bacteria multiplex LAMP assay showed 10 to 100-fold higher analytical sensitivity compared to the qPCR assay.

**Table 4:** Comparison of analytical sensitivity of the gram-positive bacteria multiplex LAMP assay and qPCR assay using ATCC strains.

A. Gram-positive bacteria										
CFU/mL	*S*. *agalactiae*				*S*. *epidermidis*			*S*. *aureus*		
	LAMP		qPCR		LAMP	qPCR		LAMP		qPCR
	Tt (SD)		Tt (SD)		Tt (SD)	Tt (SD)		Tt (SD)		Tt (SD)
1 × 10^7^	18.5 ± 0.7		24.0 ± 0.4		13.3 ± 0.4	27.2 ± 1.0		15.3 ± 0.8		27.1 ± 0.1
1 × 10^6^	20.3 ± 0.2		27.8 ± 1.5		14.0 ± 0.5	30.5 ± 0.6		17.6 ± 2.1		30.8 ± 0.3
1 × 10^5^	26.2 ± 0.6		32.3 ± 0.1		15.9 ± 0.4	32.8 ± 1.0		21.3 ± 1.1		34.2 ± 0.2
1 × 10^4^	N/A		34.2 ± 0.5		17.8 ± 1.1	35.3 ± 0.9		N/A		36.5 ± 0.9
1 × 10^3^	N/A		N/A		20.7 ± 0.7	37.6 ± 1.1		N/A		N/A
1 × 10^2^	N/A		N/A		N/A	N/A		N/A		N/A
1 × 10^1^	N/A		N/A		N/A	N/A		N/A		N/A
1 × 10^0^	N/A		N/A		N/A	N/A		N/A		N/A
0	N/A		N/A		N/A	N/A		N/A		N/A
B. Gram-negative bacteria										
CFU/mL		*P*. *aeruginosa*					*E*. *coli*			
		LAMP		qPCR			LAMP		qPCR	
		Tt (SD)		Tt (SD)			Tt (SD)		Tt (SD)	
1 × 10^7^		12.1 ± 0.4		27.4 ± 0.4			15.1 ± 0.6		19.0 ± 0.2	
1 × 10^6^		13.7 ± 0.5		30.8 ± 0.5			17.8 ± 0.8		23.0 ± 0.3	
1 × 10^5^		16.9 ± 1.1		35.1 ± 1.6			N/A		29.1 ± 0.3	
1 × 10^4^		N/A		39.9 ± 2.2			N/A		32.8 ± 1.5	
1 × 10^3^		N/A		N/A			N/A		N/A	
1 × 10^2^		N/A		N/A			N/A		N/A	
1 × 10^1^		N/A		N/A			N/A		N/A	
1 × 10^0^		N/A		N/A			N/A		N/A	
0		N/A		N/A			N/A		N/A	

LAMP: Loop-mediated isothermal amplification, qPCR: quantitative real-time PCR, ATCC: American Type Culture Collection, Tt: threshold time, SD: standard deviation, N/A: not available.

### Clinical performance of the gram-positive/negative bacteria multiplex LAMP assay

We compared the sensitivity and specificity of the LAMP assay with those of qPCR for clinical synovial fluid samples (Table 5) to validate the clinical performance of the gram-positive and gram-negative bacterial multiplex LAMP assays. The gram-positive/negative bacterial multiplex LAMP assay demonstrated 100% specificity when tested against normal joint fluid clinical samples (n = 14). For six clinical samples, including *S*. *agalactiae* (n = 1), *S*. *epidermidis* (n = 1), and *S*. *aureus* (n = 4), the gram-positive bacteria multiplex LAMP assay exhibited 100% sensitivity without cross-reaction with other target bacteria; the multiplex LAMP assay showed 100% specificity.

**Table 5:** Performance comparison between the gram-positive/negative bacteria multiplex LAMP assay and qPCR for clinical samples.

Clinical Sample		Gram-positive bacteria multiplex LAMP assay			Gram-negative bacteria multiplex LAMP assay		qPCR
		*S*. *agalactiae*	*S*. *epidermidis*	*S*. *aureus*	*P*. *aeruginosa*	*E*. *coli*	
*S*. *agalactiae* (n = 1)	P/N	1/0	0/1	0/1	0/1	0/1	1/0
	Sensitivity	100	-	-	-	-	100
	Specificity	-	100	100	100	100	-
*S*. *epidermidis* (n = 1)	P/N	0/1	1/0	0/1	0/1	0/1	1/0
	Sensitivity	-	100	-	-	-	100
	Specificity	100	-	100	100	100	-
*S*. *aureus* (n = 4)	P/N	0/4	0/4	4/0	0/4	0/4	4/0
	Sensitivity	-	-	100	-	-	100
	Specificity	100	100	-	100	100	-
Negative Sample (n = 14)	P/N	0/14	0/14	0/14	0/14	0/14	0/14
	Sensitivity	-	-	-	-	-	-
	Specificity	100	100	100	100	100	100

“P” and “N” indicate the positive and negative reactions, respectively.

LAMP: Loop-mediated isothermal amplification, qPCR: quantitative real-time PCR.

### Cross-reactivity tests

In addition to the five target bacteria for the LAMP assay developed in this study, cross-reactivity tests were performed using an additional 4 ATCC strains: *E*. *aerogenes* (ATCC 13048), *Enterococcus faecalis* (ATCC 29212), *S*. *pneumoniae* (ATCC 49619), and *K*. *pneumoniae* (ATCC 13883) (Table 6). In target bacteria, including *S*. *agalactiae*, *S*. *epidermidis*, *S*. *aureus*, *P*. *aeruginosa*, and *E*. *coli*, the gram-positive and gram-negative bacteria multiplex LAMP assay detected the target bacteria without cross-reaction with other signals targeting other target bacteria. The gram-positive and gram-negative bacteria multiplex LAMP assays exhibited no cross-reactivity for the other four infectious bacteria, including *E*. *aerogenes*, *E*. *faecalis*, *S*. *pneumoniae*, and *K*. *pneumoniae*. These results indicate that the LAMP assay can accurately detect the target bacteria without producing false-positive results, even in the presence of other bacteria.

**Table 6:** Cross-reactivity of the gram-positive/negative bacteria multiplex LAMP assay against infectious bacteria.

Test bacteria (ATCC strains)	Gram-positive bacteria multiplex LAMP assay			Gram-negative bacteria multiplex LAMP assay	
	*S*. *agalactiae*	*S*. *epidermidis*	*S*. *aureus*	*P*. *aeruginosa*	*E*. *coli*
*S*. *agalactiae*	P	N	N	N	N
*S*. *epidermidis*	N	P	N	N	N
*S*. *aureus*	N	N	P	N	N
*P*. *aeruginosa*	N	N	N	P	N
*E*. *coli*	N	N	N	N	P
*E*. *aerogenes*	N	N	N	N	N
*E*. *faecalis*	N	N	N	N	N
*S*. *pneumoniae*	N	N	N	N	N
*K*. *pneumoniae*	N	N	N	N	N

“P” and “N” indicate the positive and negative reactions, respectively.

LAMP: Loop-mediated isothermal amplification, qPCR: quantitative real-time PCR.

## Discussion

Recently, molecular diagnostic methods for PJI diagnosis were introduced to increase detection sensitivity and shorten turnaround times [33]. However, molecular diagnostic methods like real-time PCR require expensive equipment and trained personnel. Additionally, the test takes at least 2–3 h and can only be performed in the central laboratories of hospitals with all the necessary equipment [4]. Methods for identifying causative microorganisms using isothermal nucleic acid amplification have attracted renewed attention to overcome these limitations. Among the isothermal amplification methods, LAMP is most actively studied for the development of new diagnostic kits for detecting microorganisms [12, 14].

In this study, we developed a multiplex LAMP assay that can detect five bacterial species that account for many PJI-causing bacteria. We validated the performance of our developed LAMP assay by comparing it against published qPCR assays for each bacterium, whose performance has been verified, using bacterial analysis samples prepared identically. Our multiplex LAMP assay detects five bacteria in two reactions. The gram-positive assay detected three gram-positive bacteria: *S*. *epidermidis*, *S*. *agalactiae*, and *S*. *aureus*, and the gram-negative assay detected two: *P*. *aeruginosa* and *E*. *coli*. Previously, attempts were made to use LAMP to detect bacteria in orthopedic and infectious diseases; however, most of these attempts targeted a single bacterium [27, 34–36]. Multiplex LAMP assays that can simultaneously detect multiple bacteria have rarely been developed. We designed LAMP assays to enable clinicians to detect as many types of bacteria simultaneously as possible. However, like PCR and LAMP, molecular diagnostic methods using primers or probes are limited because they can only detect a predetermined number of targets. Similarly, our multiplex LAMP assay is limited to detecting only the five target bacteria we selected. We selected five bacteria based on the frequency with which they cause PJIs [22]. Although the number is limited to five, the assay is still clinically useful.

The analytical sensitivity of the LAMP assay has been reported in previous studies to be between 10^2^ and 10^5^ CFU/mL for bacteria [37–39]. In our multiplex LAMP assay, four types of bacteria except *E*. *coli* were detected at 10^3^–10^5^ CFU/mL, with analytical sensitivity values similar to those reported in previous studies. However, the analytical sensitivity of the multiplex LAMP assay for *E*. *coli* was 10^6^ CFU/mL. This may be because primer and probe efficiencies were not sufficiently high. Further research could achieve a lower analytical sensitivity. Additionally, the LODs in previous studies were all for single-plex LAMP assays, whereas ours is a multiplex LAMP assay. Therefore, we inferred that, overall, the analytical sensitivity of our assay was higher than that reported in previous studies [40]. The sensitivity to each target does not deteriorate when LAMP is developed as a multiplex assay, as with real-time PCR [40, 41]. In our study, the analytical sensitivity was approximately 10–10^2^ times lower when each type of bacterium was individually tested in a single-plex assay than in a multiplex assay. However, we postulated that for diagnosing PJI, it would be helpful to detect as many types of bacteria as possible in a single reaction by increasing the analytical sensitivity using a multiplex, and we developed a multiplex assay.

We compared our multiplex LAMP assay, including analytical sensitivity, with a qPCR assay reported in the literature; among the PCR-based molecular assays, there is no widely used commercial real-time PCR kit for PJI diagnosis. To compare the analytical sensitivity, we designed a single-plex qPCR assay targeting each of the five bacteria and compared it with the multiplex LAMP assay developed in this study. Using our LAMP assay, gram-positive bacteria, such as *S*. *agalactiae* and *S*. *aureus*, had an analytical sensitivity approximately ten times higher than that of qPCR, whereas *S*. *epidermidis* had the same analytical sensitivity. Additionally, *P*. *aeruginosa* had an analytical sensitivity ten times higher than qPCR among gram-negative bacteria, whereas *E*. *coli* had an analytical sensitivity approximately 100 times higher. According to a previous study, the analytical sensitivity of LAMP was approximately 100 times lower than that of conventional PCR and 10–100 times higher than that of real-time PCR [39]. Therefore, the multiplex LAMP assay developed in this study showed a consistent analytical sensitivity difference compared to qPCR. Additionally, it should be noted that our analytical sensitivity analysis compared multiplex LAMP with single-plex qPCR; the analytical sensitivity difference may be even greater.

We tested our multiplex LAMP assay for cross-reactivity by adding four additional ATCC strains, *E*. *aerogenes*, *E*. *faecalis*, *S*. *pneumoniae*, and *K*. *pneumoniae* to determine whether it would produce a positive reaction to bacteria other than the five targeted bacteria. The test results showed that the assay did not produce a positive reaction to any bacteria other than the original target bacteria, indicating excellent specificity. In a clinical performance evaluation using synovial fluid from actual patients, the assay showed 100% sensitivity and specificity compared to the culture results. Although the clinical sample size was small, these results indicate that the performance of the gram-positive/negative bacterial multiplex LAMP assay is similar to that of the qPCR assay for detecting bacteria in joint synovial fluid clinical samples. Therefore, our gram-positive/negative bacterial multiplex LAMP assay is a reliable alternative for detecting these pathogens in clinical samples.

In our study, we developed a LAMP test that showed significantly faster results compared to the qPCR test. The LAMP test took only 30 minutes to complete, while the qPCR test took approximately 120 minutes. The LAMP test’s rapid turnaround time is attributed to its isothermal amplification process, which eliminates the need for temperature cycling steps required in PCR. This makes the LAMP test more efficient and faster. Furthermore, the LAMP test only requires a heat block to maintain a constant temperature, whereas the qPCR requires a costly thermocycler. This makes the LAMP test more accessible and cost-effective for diagnostic applications.

The types of bacteria that can be detected by the multiplex LAMP assay developed in this study are mostly consistent with those commonly found to cause septic arthritis or osteomyelitis [42–45]. Therefore, our assay can diagnose bacterial septic arthritis and osteomyelitis and is a high-throughput assay that can diagnose PJI and other orthopedic infections.

This study had several limitations. First, this study focused on developing a multiplex LAMP assay for diagnosing PJI and evaluating its analytical performance using ATCC standard strains. The clinical performance evaluation used a few clinical specimens. Most were negative specimens, and only a few positive specimens were used. Therefore, further studies using sufficient clinical specimens are required to confirm the clinical utility of the LAMP assay developed in this study. Second, our LAMP assay can only detect target bacteria that bind to primers and probes, and the number of multiplexing reactions that can be performed is limited. Therefore, the multiplex LAMP assay developed in this study cannot detect bacteria other than the five selected targets or other types of microorganisms that rarely cause PJI, such as fungi and mycobacteria. Third, we did not include an internal control in this study. Adding an internal control in future research is crucial to ensure more accurate and reliable results. Fourth, this study highlights LAMP’s efficiency and simplicity over PCR, but we used the same equipment typically required for PCR. Thus, this study may not fully show LAMP’s potential for less complex and more affordable operation. However, recent advances have led to the development and commercial availability of cost-effective, portable isothermal amplification devices capable of four-channel multiplexing (https://www.gencurix.com/eng/sub_2_8.php). Consequently, future studies evaluating LAMP kits with these portable devices could fully leverage LAMP’s advantages, demonstrating its cost-effectiveness, simplicity, and rapid operation.

In conclusion, the multiplex LAMP assay developed in this study can rapidly detect five bacterial species with high prevalence among the causative bacteria of bacterial PJI with excellent sensitivity and specificity in less than 1 h. Thus, the multiplex LAMP assay may be helpful in the early diagnosis of PJI.

## Supporting information

S1 Raw data (XLSX)
